# Subacute Post-Traumatic Ascending Myelopathy (SPAM) in a Spinal Cord Injured Patient - a Rare Presentation: A Case Report

**DOI:** 10.5704/MOJ.2103.022

**Published:** 2021-03

**Authors:** NK Kassim, MH Hanafi, AH Ibrahim, N Hasnan

**Affiliations:** 1 Basic Science and Oral Biology Unit, Universiti Sains Malaysia, Kubang Kerian, Malaysia; 2 Rehabilitation Medicine Unit, Universiti Sains Malaysia, Kubang Kerian, Malaysia; 3 Department of Rehabilitation Medicine, University of Malaya, Kuala Lumpur, Malaysia

**Keywords:** myelopathy, hypotension, spinal cord injury

## Abstract

The optimisation of blood pressure management is critical in managing hypotensive episodes in patients with spinal cord injury. Improper handling of this preventable factor will negatively impact the patient recovery prognosis. A 42-year-old man was admitted for a complete spinal cord injury after fell from height. He developed subacute neurological deterioration unrelated to the mechanical instability but due to multiple episodes of hypotension occurring one month after the initial injury. After proper management of blood pressure, his deterioration was halted and no further progression. Spinal cord haemodynamics play an important role in mediating the onset of subacute post-traumatic ascending myelopathy. Better education and awareness on Subacute Post-traumatic Ascending Myelopathy (SPAM) especially to the junior healthcare providers are important to hinder this rare but avoidable condition.

## Introduction

New neurological deterioration after initial spinal cord injury which is not attributable to spinal column instability is rare^1^. The rise of the level of the lesion or myelopathy during the first few hours and days are attributed to the oedema and other secondary changes that affect the one or two segments near to the injured site^1^.

In between these acute and late complications, a rare subacute neurological deterioration that cannot be attributed to mechanical instability can occur between Day One (1) to the fourth week after the initial injury. This rare form of presentation is known as Subacute Post-traumatic Ascending Myelopathy (SPAM)^2^. Although several hypotheses have been postulated, the exact mechanism of this condition still unknown. Furthermore, there is a dearth in the literature describing ascending weakness following multiple hypotensive episodes in the subacute phase of spinal cord injury.

## Case Report

**Fig. 1:**
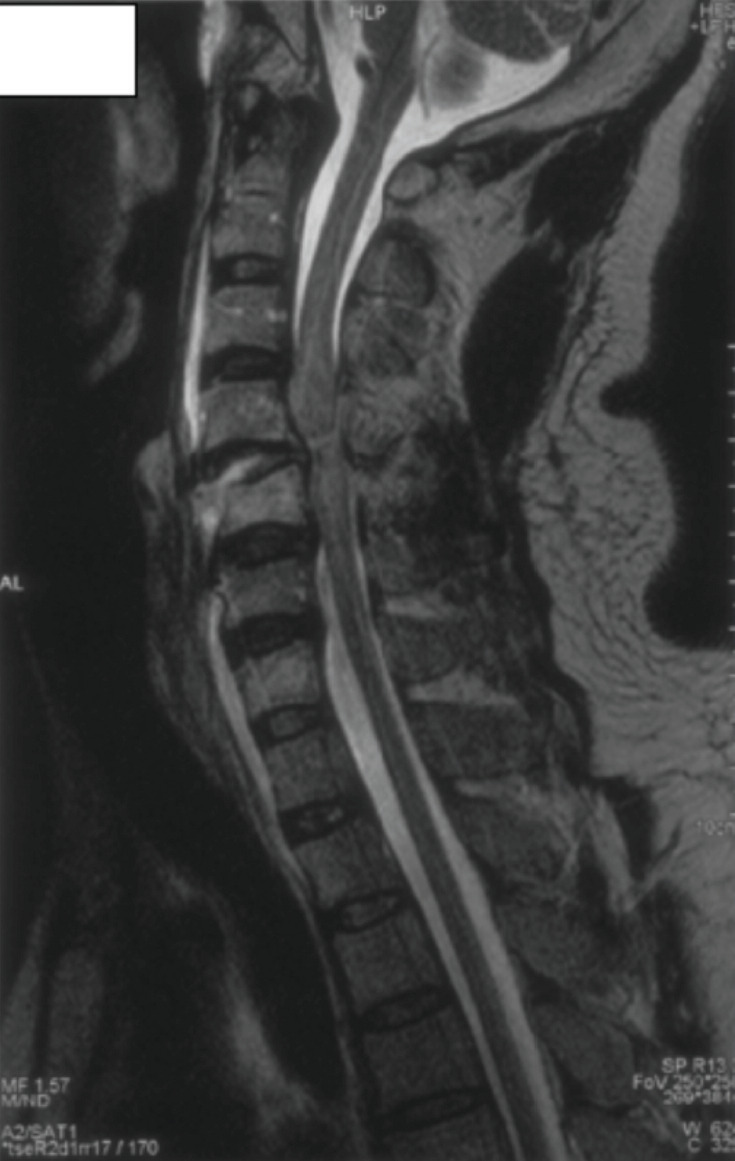
Complete transection of C5 level of spinal cord and cord oedema (Day 1 MRI after admission).

A 42-year-old male sustained fracture of the C5 vertebral body and anterolisthesis of C4-C5 vertebra following a fall from height (15m). This traumatic fall resulted in marked stenosis of the central canal. Neurological assessment at admission was C4 AIS A. Initial magnetic resonance imaging (MRI) demonstrated complete transection of C5 level of spinal cord and cord oedema from C4 disc level to C7 vertebral body level (Fig. 1). There was no neurological problem before this event.

The spinal fracture-dislocation was surgically fixed at Day 3 post-injury using posterior decompression with lateral mass fusion of C3-C6 method. The surgery successfully stabilised the fracture with resulting good alignment and better spinal canal arrangement. Post-operatively, there was no further deterioration of AIS level compared to the pre-operative condition. The patient was transferred to the rehabilitation ward for adaptive living conditioning training as a tetraplegic patient once his condition improved and the orthopaedic surgical team assured of the spinal stability established after the surgery. His Spinal Cord Independence Measure III (SCIM III) level was 16/100 (Selfcare 7, Respiration and Sphincter 4, Mobility 5) after three weeks of inpatient rehabilitation.

However, at Day 30 post-injury, he started to complain of frequent dizziness related to postural changes during rehabilitation therapy. His supine blood pressure ranged from 93/47 to 127/68mmHg, while his 30° head-up blood pressure ranged from 62/42 to 78/48mmHg. His blood pressure lability was attributed to autonomic dysfunction and as the condition was not improved and his consciousness worsened, he required regular intravenous inotrope infusion to prevent circulatory collapse.

**Fig. 2:**
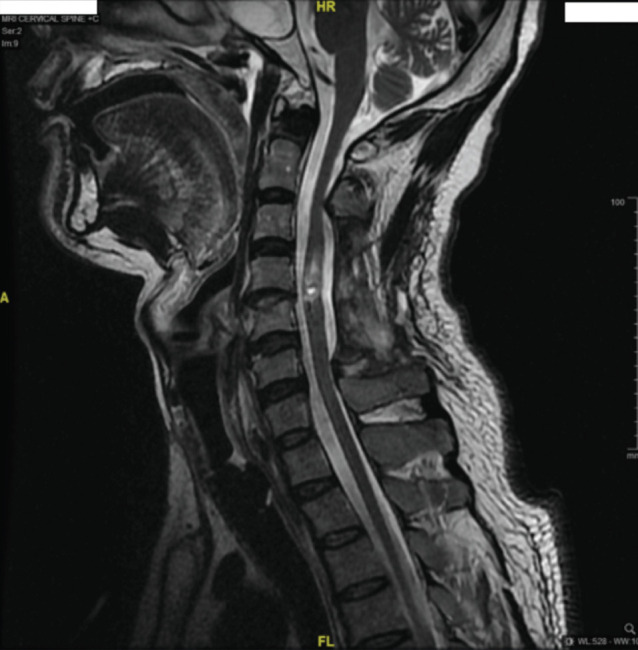
Another MRI of cervical and thoracic spine at day 34 post injury showed an increased T2 weighted signal from C4 to C5 with cord.

A few days later he started to note ascending level of numbness to his head which he described as insidious in nature. Neurological examination confirmed that his sensory level had ascended to C2 level and he was now neurologically C2 AIS A. An MRI of cervical and thoracic spine at Day 34 post injury showed an increase of T2 weighted signal from C4 to C5 with cord edema. The cord swelling was prominent at level C4 to C6 without spinal canal stenosis. There was no gadolinium enhancement which indicated that there was no new breakdown of the blood-brain barrier and inflammation (Fig. 2). His weekly SCIM III scoring at this time was only 4 over 100 (Selfcare 0, Respiration and Sphincter 4, Mobility 0).

Concurrent to this episode, although there were no clinical signs of infection, he was suspected to have subclinical sepsis from the urine culture (0-2 pus cell per high power field and gram negative Klebsiella pneumoniae was noted) and an increase in white blood cells count (13 x 109/L) which could have compounded the hypotensive episodes. Repeated blood culture then cleared him of sepsis after a full course of intravenous antibiotics. On-the-bed postural reconditioning management (compression stockings, graded conditioning on tilt table, and strict vital signs and hydration charting to prevent another postural hypotension) was restarted once he was stable to participate in the therapy again. Diagnosis of Spontaneous Post-traumatic Ascending Myelopathy was made after ruling out all other causes of worsening AIS level.

He was then discharged from the ward at Day 50; SCIM III was 32 over 100 (Selfcare 7, Respiration and Sphincter 20, Mobility 5). His AIS level during discharge was C2 AIS A.

During his first clinic appointment at three months post-injury (Day 91), his sensory level improved to C3 level, his autonomic function much improved and he was able to sit at 90° without dizziness and participated well in his rehabilitation therapies.

## Discussion

Subacute Post-traumatic Ascending Myelopathy (SPAM) was first described by Frankel in 1969 and he estimated that this condition affects nearly 1% of spinal cord injury patients^1^. Numerous hypotheses have been suggested which include secondary injury, arterial or venous blockage, fibrocartilage embolisation, apoptosis, infection, autoimmune response and alteration in CSF drainage^2^.

Postural hypotension is defined, by The Consensus Committee of the American Autonomic Society and the American Academy of Neurology (1996) as a decrease in systolic blood pressure of 20mmHg or more, or in diastolic blood pressure of 10mmHg or more, upon the assumption of an upright posture from a supine position, regardless of whether symptoms occur. Risk of experiencing postural hypotension is greater in patients with higher spinal cord lesions, and for this reason it is more common in individuals with tetraplegia3. Postural hypotension in spinal cord injury is due to multiple related causes which include sympathetic nervous system dysfunction, altered baroreflex function, lack of skeletal muscle pumping activity, cardiovascular deconditioning, and altered salt and water balance^3^. In this patient, postural hypotension was recognised as one of the factors that lead to impairment of his blood pressure regulation during the subacute stage.

Blood pressure lability in this case was also attributed to autonomic dysregulation that commonly occur in patients with high-level spinal cord injuries. The ascending pattern of neurological deterioration was presumed to be due to ischemia of the vulnerable spinal arteries in the cervical region during multiple insults from the hypotensive episodes. Despite numerous central arteries in the cervical cord, it was suggested that there is a high ischemic vulnerability of the cervical spinal cord at level C2 - C3^4^.

The postulated hypotheses of etiological mechanism of SPAM include alteration of cerebrospinal fluid (CSF) circulation, Great Artery of Adamkiewicz (GAA) thrombosis, venous thrombosis and congestive ischemia of the spinal cord vessels, infection, apoptosis secondary to spinal cord injury and hypotensive ischemia5. The ascending myelopathy pathophysiology most likely multifactorial which include the inflammation, secondary injury, vascular insufficiency, subsequent post-operative trauma (i.e. during positioning or handling of patient), haematomyelia or even the healing process^5^. Improper management of blood pressure during acute and subacute stage can reduce the perfusion and hence lead to further ischemic insult. Frequent unsupervised prolonged sitting position might be one of the factors for the orthostatic pressure drop which lead to SPAM in this case.

## Conclusion

In summary, SPAM is a rare, preventable, life-threatening consequence of spinal cord injury. If left untreated, it can later hinder the potential neurological and functional recovery as well as rehabilitation potential in traumatic spinal cord injured patients. Junior and inexperience medical practitioners should not take light of this condition as the rehabilitation prognosis is poorer if the AIS spinal level is higher.
